# Differences Between Oncological and Non-oncological Patients Referred to an In-Hospital Palliative Care Support Team: A Retrospective Cohort Study in Northern Portugal

**DOI:** 10.7759/cureus.115983

**Published:** 2026-09-09

**Authors:** Micaela José N Sousa, Luís Reis, João Bragada, Ana I Gonçalves, Eliana Rocha, Teresa G Rocha

**Affiliations:** 1 Internal Medicine, Unidade Local de Saúde do Nordeste, Bragança, PRT; 2 Epidemiology, Infection Prevention and Control, and Antimicrobial Resistance Service, Unidade Local de Saúde do Nordeste, Bragança, PRT; 3 Palliative Care, Unidade Local de Saúde do Nordeste, Bragança, PRT

**Keywords:** cancer, end-of-life care, hospital palliative care support team, non-cancer, palliative care, palliative care access, referral patterns

## Abstract

Background: Although specialist palliative care has traditionally focused on patients with advanced cancer, the growing burden of chronic non-oncological diseases has highlighted the need for equitable access to palliative care services. Nevertheless, non-oncological patients continue to experience delayed referral and underutilization of specialist palliative care. This study aimed to compare the characteristics, referral patterns, and clinical outcomes of oncological and non-oncological patients referred to an in-hospital palliative care support team (PCST).

Methods: We conducted a retrospective observational study including all adult patients referred to an in-hospital PCST during 2024. Patients were classified as oncological or non-oncological according to their primary diagnosis. Oncological patients were defined as those with active malignant neoplasm or advanced cancer-related disease documented as the underlying condition prompting referral to the palliative care team.

Demographic characteristics, referral timing, time from referral to specialist assessment, in-hospital mortality, and transfer to a specialized palliative care unit were compared between groups. Continuous variables were analyzed using the Mann-Whitney U test, while categorical variables were compared using the chi-square or Fisher's exact test.

Results: A total of 204 patients were referred to the PCST during the study period. After excluding 8 patients (3.9%) with missing information regarding the underlying diagnosis, 196 patients were included in the comparative analysis, comprising 106 oncological patients (54.1%) and 90 non-oncological patients (45.9%). Non-oncological patients were significantly older than oncological patients (mean age 84.1 vs. 76.1 years, p < 0.001). In-hospital mortality was significantly higher among non-oncological patients (44/90 (48.9%) vs. 21/106 (19.8%), p < 0.001), whereas transfer to a specialized palliative care unit was more frequent among oncological patients (45/106 (42.5%) vs. 15/90 (16.7%), p < 0.001).

Conclusions: Non-oncological patients referred to the in-hospital PCST were older, experienced worse hospital outcomes, and had lower access to specialized palliative care units than oncological patients. The comparable response time following referral suggests that disparities arise primarily in patient identification and referral rather than in the delivery of specialist palliative care. Although non-oncological patients had a longer median interval between hospital admission and referral, this difference did not reach statistical significance, and therefore delayed referral cannot be considered a definitive explanation for the observed disparities. These findings suggest that differences between the two groups may reflect differences in disease trajectory, clinical complexity, and patterns of recognition of palliative care needs.

## Introduction

Background

The global increase in life expectancy, coupled with advances in the treatment of chronic diseases, has resulted in a growing number of patients living with advanced progressive illnesses requiring palliative care. The World Health Organization (WHO) recognizes palliative care as an essential component of integrated health services and emphasizes its applicability across a broad range of life-threatening and chronic diseases, rather than cancer alone [1]. The need for palliative care is expected to continue increasing as populations age, and the burden of non-communicable diseases rises [1-3].

Although palliative care historically developed within oncology, contemporary definitions emphasize that it should be available to patients with serious illness irrespective of diagnosis [2]. Patients with advanced non-oncological diseases--including heart failure, chronic respiratory disease, chronic kidney disease, dementia and neurodegenerative disorders--may experience substantial symptom burden, functional decline and psychosocial distress, with palliative care needs comparable in many respects to those of patients with advanced cancer [1,3-5].

Despite this broader conceptual framework, access to specialist palliative care remains uneven. Patients with cancer generally have more established referral pathways and are more likely to receive specialist palliative care than patients with non-oncological diseases [6,7]. This disparity has been described across different healthcare systems and is particularly relevant in hospital settings, where referral to specialist palliative care may be influenced by the recognition of an appropriate clinical trigger by the treating team.

Differences in disease trajectories may contribute substantially to this phenomenon. Advanced cancer often follows a comparatively recognizable trajectory of progressive deterioration, whereas chronic organ failure and neurodegenerative diseases may be characterized by prolonged periods of relative stability interrupted by recurrent episodes of acute deterioration [7,8]. These less predictable trajectories create considerable prognostic uncertainty and may make it more difficult for clinicians to determine when specialist palliative care should be introduced.

Murray et al. described distinct illness trajectories associated with cancer, organ failure and frailty, emphasizing that patients with organ failure may experience repeated episodes of deterioration and partial recovery before a final decline [7]. Similarly, Lunney et al. demonstrated that patterns of functional decline differ substantially according to underlying disease, with non-cancer populations frequently experiencing prolonged periods of disability and fluctuating clinical status [8]. These differences may contribute to delayed recognition of palliative care needs and consequently to later referral.

Recognizing patients who may benefit from palliative care before terminal deterioration is therefore an important clinical challenge. Boyd and Murray proposed a structured approach based on identifying key transitions in the course of advanced illness rather than relying exclusively on prognostic estimates [9]. Several clinical tools have subsequently been developed to support this process. The Supportive and Palliative Care Indicators Tool (SPICT) was developed to help clinicians identify patients at risk of deterioration and death who may benefit from a palliative approach [10], while the NECPAL instrument was developed to identify patients with chronic conditions who may have unmet palliative care needs [11].

Specialist palliative care teams may play an important role in addressing these needs within acute hospitals. Hospital-based palliative care support teams (PCSTs) provide specialist input for complex symptom management, communication and decision-making, advance care planning and coordination of transitions of care. Professional organizations increasingly recommend integration of specialist palliative care alongside disease-directed treatment, particularly for patients with advanced cancer and high symptom or psychosocial burden [12]. Randomized clinical trials have demonstrated that early palliative care integration can improve quality of life, symptom control and end-of-life outcomes, while potentially reducing inappropriate or burdensome interventions [13,14]. Systematic reviews have similarly reported beneficial effects of specialist palliative care on patient-centred outcomes [15].

The integration of specialist and generalist palliative care has consequently been proposed as a sustainable model for meeting the growing demand associated with serious illness [16]. Importantly, this model requires access to specialist services to be determined by the complexity of patients' needs rather than by the presence of a particular diagnosis. However, evidence suggests that implementation remains heterogeneous across healthcare systems, with substantial variation in referral pathways and access among patients with non-oncological diseases [17,18].

The growing burden of serious health-related suffering further emphasizes the importance of addressing these disparities. Global projections indicate a substantial increase in the number of people requiring palliative care over the coming decades, driven largely by population ageing and the increasing prevalence of chronic non-communicable diseases [3-5]. Ensuring equitable access to specialist palliative care is therefore both a clinical and an organizational priority.

Although differences between oncological and non-oncological patients have been documented internationally, evidence from Portuguese hospital-based palliative care services remains limited. In particular, there is a need to better characterize whether differences in age, referral timing, mortality, and access to specialized inpatient palliative care persist within the same hospital-based service.

Understanding these patterns may help identify potential barriers to timely referral and inform strategies aimed at achieving more equitable access to specialist palliative care.

Objectives

The primary objective of this study was to compare demographic characteristics, referral timing, and hospital outcomes between oncological and non-oncological patients referred to an in-hospital PCST during a one-year period.

The secondary objectives were to identify factors associated with in-hospital mortality, to determine whether oncological disease independently predicts mortality after adjustment for relevant clinical and demographic variables, and to compare access to specialized palliative care units between oncological and non-oncological patients.

## Materials and methods

Study design

This retrospective observational cohort study included all consecutive adult patients referred to the in-hospital PCST of Northeast Local Health Unit, a Portuguese secondary care hospital, between 1 January and 31 December 2024.

The study was conducted in accordance with the Strengthening the Reporting of Observational Studies in Epidemiology (STROBE) recommendations for observational studies [19].

Study setting

The hospital provides secondary and differentiated healthcare to a predominantly rural population and includes an in-hospital PCST composed of physicians and nurses with specialist training in palliative medicine. The PCST provides consultation for hospitalized adult patients referred by any medical or surgical specialty.

Study population

All consecutive adult patients referred to the PCST during the study period were eligible. Patients were classified into two groups according to the underlying disease: oncological disease and non-oncological disease. Patients without a valid disease classification were excluded from comparative analyses but remained included in the overall description of referrals.

Definition of oncological and non-oncological patients

Patients were classified into two groups according to the underlying condition documented at the time of referral to the in-hospital PCST. Oncological patients were defined as those with an active malignant neoplasm or advanced cancer-related disease documented as the underlying condition prompting referral to the palliative care team. Patients with a previous history of cancer but without evidence of active or advanced malignant disease at the time of referral were not classified as oncological solely on the basis of a remote cancer diagnosis.

Non-oncological patients were defined as patients with advanced, progressive or life-limiting non-malignant diseases for whom palliative care was requested, including advanced organ failure, neurodegenerative disease, dementia, frailty and other serious chronic conditions. Patients for whom the oncological/non-oncological classification could not be established from the available clinical records were excluded from the comparative analysis.

Data collection

Clinical information was extracted retrospectively from the institutional electronic referral database. The following variables were collected: age; sex; underlying diagnosis; referring specialty; hospital admission date; referral date; first specialist assessment date; transfer to a specialized palliative care unit; hospital outcome. Derived variables included: interval between hospital admission and referral; interval between referral and first specialist assessment.

Outcomes

The primary outcome was the comparison between oncological and non-oncological patients regarding demographic characteristics, referral timing, in-hospital mortality, and/or transfer to a specialized palliative care unit. The secondary outcome was identifying independent predictors of in-hospital mortality using multivariable logistic regression.

Statistical analysis

Continuous variables were assessed for distribution and are presented as means and standard deviations or medians and interquartile ranges, as appropriate. Categorical variables are presented as frequencies and percentages. Comparisons between groups were performed using the Mann-Whitney U test for continuous variables and the chi-square test for categorical variables.

Variables considered clinically relevant or statistically significant in univariate analyses were entered into a multivariable logistic regression model to identify factors independently associated with in-hospital mortality. Wald χ² statistic was used to test the null hypothesis that the regression coefficient equals zero. Results are reported as adjusted odds ratios (ORs) with corresponding 95% confidence intervals (95% CIs).

A two-sided p-value <0.05 was considered statistically significant. Statistical analyses were performed using IBM SPSS Statistics (IBM Corp., Armonk, NY).

Ethical considerations

The study was conducted in accordance with the principles of the Declaration of Helsinki and applicable national legislation regarding retrospective observational studies. Patient confidentiality was preserved throughout the study by anonymizing all extracted data before analysis. Approval was obtained from the local ethics committee prior to data collection, in accordance with institutional requirements.

## Results

Baseline characteristics

During the study period, 204 consecutive patients were referred to the in-hospital PCST. Eight patients (3.9%) were excluded from the comparative analyses because of missing information regarding the underlying diagnosis. Consequently, 196 patients were included in the final analysis, comprising 106 patients with oncological disease (54.1%) and 90 patients with non-oncological disease (45.9%). The demographic characteristics, referral patterns, and clinical outcomes are summarized in Table 1.

**Table 1:** Comparison of demographic characteristics, referral timing and clinical outcomes between oncological and non-oncological patients referred to the In-Hospital Palliative Care Support Team. Continuous variables were compared using the Mann-Whitney U test; categorical variables were compared using the Pearson chi-square (χ²) test.*p < 0.05 was considered statistically significant. PCU = palliative care unit; U = Mann-Whitney U statistic; χ² = Pearson chi-square statistic.

Characteristic	Oncological (n=106)	Non-oncological (n=90)	Test statistic	p-value
Mean age (years)	76.1	84.1	U = 2880.0	<0.001*
Median age (years)	78	87	U = 2880.0	<0.001*
Male sex, n (%)	55 (51.9%)	37 (41.1%)	χ² = 2.543	0.111
Female sex, n (%)	51 (48.1%)	53 (58.9%)	χ² = 2.543	0.111
Median admission-to-referral interval (days)	5	7	U = 4038.5	0.064
Median referral-to-assessment interval (days)	1	1	U = 3878.0	0.237
In-hospital mortality, n (%)	21 (19.8%)	44 (48.9%)	χ² = 18.567	<0.001*
Transfer to specialized PCU, n (%)	45 (42.5%)	15 (16.7%)	χ² = 15.237	<0.001*

Patients with non-oncological diseases were significantly older than those with oncological diseases (mean age 84.1 vs. 76.1 years; median age 87 vs. 78 years; p < 0.001). The median interval between hospital admission and referral to the PCST tended to be longer among non-oncological patients (7 vs. 5 days), although the difference did not reach statistical significance (p = 0.064).

Following referral, both groups were assessed equally rapidly by the PCST, with a median interval of one day between referral and first specialist assessment (p = 0.237).

Marked differences were observed in hospital outcomes. In-hospital mortality was significantly higher among non-oncological patients (44/90 (48.9%) vs. 21/106 (19.8%), p < 0.001), whereas oncological patients were significantly more likely to be transferred to a specialized palliative care unit (45/106 (42.5%) vs. 15/90 (16.7%), p < 0.001).

These findings suggest that differences between the two populations occur mainly before referral to specialist palliative care rather than in the response provided by the PCST.

Factors associated with in-hospital mortality

Univariate logistic regression analysis identified both age and underlying disease type as significant predictors of in-hospital mortality (Table 2). Patients with oncological disease had substantially lower odds of dying during hospital admission than patients with non-oncological disease. Increasing age was associated with a progressively higher probability of in-hospital mortality. Sex and the interval between admission and referral were not significantly associated with mortality.

**Table 2:** Univariate logistic regression analysis of factors associated with in-hospital mortality. Univariate logistic regression models were fitted for each predictor separately. The Wald χ² statistic was used to test the null hypothesis that the regression coefficient equals zero. *p < 0.05 was considered statistically significant. OR = odds ratio; CI = confidence interval; χ² = Wald chi-square statistic.

Variable	OR	95% CI	Wald χ²	p-value
Oncological disease	0.26	0.14–0.49	17.645	<0.001*
Age (per year)	1.05	1.02–1.08	9.359	0.002*
Male sex	0.9	0.49–1.63	0.129	0.72
Admission-to-referral interval (per day)	0.99	0.97–1.01	0.865	0.352

Multivariable logistic regression

Variables demonstrating clinical relevance or statistical significance in univariate analyses were entered into a multivariable logistic regression model. After adjustment for age, sex, and admission-to-referral interval, oncological disease remained independently associated with a significantly lower risk of in-hospital mortality (adjusted OR 0.30; 95% CI 0.15-0.59; p = 0.001). Increasing age remained independently associated with mortality (adjusted OR 1.03 per year; 95% CI 1.00-1.06; p = 0.048). Neither sex nor admission-to-referral interval independently predicted mortality, as shown in Table 3.

**Table 3:** Multivariable logistic regression analysis of independent predictors of in-hospital mortality. A multivariable logistic regression model included age, sex, underlying disease type and admission-to-referral interval. The Wald χ² statistic was used to test the null hypothesis that each adjusted regression coefficient equals zero. *p < 0.05 was considered statistically significant. OR = odds ratio; CI = confidence interval; χ² = Wald chi-square statistic.

Variable	Adjusted OR	95% CI	Wald χ²	p-value
Oncological disease	0.3	0.15–0.59	12.079	0.001*
Age (per year)	1.03	1.00–1.06	3.911	0.048*
Male sex	1.27	0.66–2.47	0.508	0.476
Admission-to-referral interval (per day)	0.98	0.96–1.01	1.443	0.23

## Discussion

The present study identified significant differences between oncological and non-oncological patients referred to an in-hospital PCST, despite comparable time from referral to specialist assessment. Non-oncological patients were substantially older and had markedly higher in-hospital mortality. Although the interval from hospital admission to referral tended to be longer in this group, the difference was not statistically significant. Therefore, delayed referral cannot be established as the primary explanation for the observed differences. Instead, these findings may reflect differences in disease trajectories, clinical complexity, and recognition of palliative care needs.

The higher age of non-oncological patients is consistent with the epidemiology of advanced chronic disease and the demographic transition affecting healthcare systems worldwide [3-5]. The burden of serious health-related suffering is expected to increase substantially, driven predominantly by population ageing and the growing prevalence of chronic non-communicable diseases [4,5]. Advanced heart failure, chronic respiratory disease, dementia and other progressive non-oncological conditions consequently represent an increasingly important population for specialist palliative care [1,3-5].

Age alone, however, does not explain the differences observed. After adjustment for age, sex, and admission-to-referral interval, oncological disease was associated with lower odds of in-hospital mortality. This association may nevertheless reflect unmeasured differences in disease severity, frailty, and comorbidity. Disease trajectory, referral practices, access to specialist services, and clinical decision-making may also contribute to the poorer outcomes observed among non-oncological patients. This is clinically relevant because contemporary palliative care frameworks emphasize that access should be based on the severity and complexity of patients' needs rather than diagnosis alone [1,2].

The tendency towards a longer interval between admission and referral among non-oncological patients, although not statistically significant, is consistent with challenges in the timely recognition of palliative care needs described in the international literature [6]. Referral pathways have historically been more clearly established in oncology, often supported by disease-specific trajectories and recognizable clinical triggers. In contrast, chronic non-oncological diseases frequently follow less predictable trajectories, with prolonged periods of relative stability interrupted by acute deterioration and partial recovery, making prognostication more challenging [7,8]. Murray et al. described distinct illness trajectories associated with cancer, organ failure and frailty, while Lunney et al. demonstrated substantial differences in patterns of functional decline across diseases [7,8]. In this context, increasing symptom burden, functional deterioration and recurrent hospitalizations may be more useful indicators of palliative care need than precise prognostic estimates [9].

Structured identification tools may help address this challenge. The Supportive and Palliative Care Indicators Tool (SPICT) and NECPAL were developed to identify patients with advanced chronic disease who may benefit from a palliative approach [10,11]. Their integration into routine clinical practice could reduce reliance on subjective prognostic judgement and facilitate earlier recognition of unmet palliative care needs among non-oncological patients.

The substantially higher in-hospital mortality among non-oncological patients is another important finding. Almost half of these patients died during hospitalization, compared with approximately one-fifth of oncological patients, and this difference persisted after multivariable adjustment. Several factors may contribute. Non-oncological patients may reach specialist palliative care after more advanced clinical deterioration, limiting opportunities for symptom optimization, advance care planning, and discharge planning. Prognostic uncertainty may further contribute to therapeutic inertia, as clinicians managing chronic organ failure, dementia, and other progressive conditions may find it difficult to determine when disease-directed treatment should be complemented by predominantly palliative management [7-9]. Frailty and multimorbidity may also play an important role, although these were not directly measured in the present study [3-5].

Importantly, the interval from referral to specialist assessment was comparable between groups. Once referred, the PCST therefore responded with similar speed regardless of diagnosis, suggesting that disparities arise predominantly before specialist involvement rather than from differences in the delivery of specialist palliative care. Expanding specialist capacity alone is unlikely to eliminate these disparities if non-oncological patients remain less readily identified and referred.

Hospital-based palliative care should consequently be approached as an integrated model in which specialist teams support generalist clinicians caring for patients with complex palliative care needs [16-18]. European experience demonstrates substantial heterogeneity in models of integrated palliative care, underscoring the importance of organizational structures that facilitate timely identification and referral across diagnoses [17]. More broadly, palliative care should be integrated into the management of serious illness rather than restricted to the final phase of life [18].

From a clinical and organizational perspective, these findings support a shift towards needs-based rather than diagnosis-based referral, consistent with the WHO definition of palliative care and contemporary international consensus [1,2]. Standardized referral criteria, clinician education, systematic screening, and integration of tools such as SPICT and NECPAL into clinical workflows or electronic health records may improve recognition of palliative care needs, particularly in internal medicine and other settings caring for older patients with multimorbidity [10,11].

Overall, improving equity in specialist palliative care requires more than expanding specialist services. The key challenge may lie in recognizing palliative care needs earlier across the hospital, particularly among patients with advanced non-oncological disease. Earlier identification may create greater opportunities for symptom management, communication, advance care planning, and appropriate discharge planning before irreversible deterioration occurs.

Strengths and limitations

The present study has several strengths. First, it included all consecutive referrals to the in-hospital PCST during a complete calendar year, minimizing selection bias and reflecting routine clinical practice. Second, the inclusion of both oncological and non-oncological patients allowed direct comparison of referral patterns and outcomes within the same healthcare setting. Third, the use of multivariable logistic regression strengthened the analysis by accounting for potential confounding factors such as age and sex.

This study has several limitations that should be considered when interpreting its findings. First, its retrospective, single-centre observational design limits the ability to establish causal relationships. In particular, the finding of an adjusted OR of 0.30 for oncological disease should not be interpreted as evidence that having cancer is a protective factor against mortality. Rather, it indicates a statistical association between oncological status and lower odds of in-hospital mortality within this specific cohort, after adjustment for the variables included in the model. This association may reflect residual confounding, differences in disease trajectories, case-mix, referral patterns, treatment intensity, functional status, comorbidity burden, or other unmeasured clinical factors. Therefore, the results should not be interpreted as suggesting that cancer itself reduces the risk of death.

Second, several potentially relevant prognostic variables, including frailty, functional status, comorbidity burden, disease severity, performance status, and reason for referral, were not systematically available for adjustment. These factors may differ substantially between oncological and non-oncological populations and could partly explain the observed differences in mortality. The relatively small sample size also limits the statistical power and precision of the multivariable model. Finally, because the study was conducted in a single Portuguese hospital and included only patients referred to an in-hospital PCST, the findings may not be generalizable to other hospitals, healthcare systems, or patients who were not referred for specialist palliative care.

## Conclusions

This study identified important differences between oncological and non-oncological patients referred to an in-hospital PCST. Non-oncological patients were older and had substantially higher in-hospital mortality, whereas oncological patients were more frequently transferred to a specialized palliative care unit. These findings suggest that patients with advanced non-malignant disease may be referred later in the course of their illness and may have greater unmet palliative care needs.

Improving equity in palliative care requires a shift from diagnosis-based to needs-based referral. Earlier identification of patients with advanced non-oncological disease, supported by standardized referral criteria and systematic screening tools, may facilitate timely specialist involvement and improve the coordination of symptom management, goals-of-care discussions, and end-of-life planning.
